# Adalimumab Reduces Photoreceptor Cell Death in A Mouse Model of Retinal Degeneration

**DOI:** 10.1038/srep11764

**Published:** 2015-07-14

**Authors:** Cristina Martínez-Fernández de la Cámara, Alberto M. Hernández-Pinto, Lorena Olivares-González, Carmen Cuevas-Martín, María Sánchez-Aragó, David Hervás, David Salom, José M. Cuezva, Enrique J. de la Rosa, José M Millán, Regina Rodrigo

**Affiliations:** 1 Grupo de Biomedicina Molecular, Celular y Genómica, Instituto de Investigación Sanitaria La Fe, Valencia, Spain; 2 3D Lab (Desarrollo, Diferenciación & Degeneración), Departamento de Medicina Celular y Molecular, Centro de Investigaciones Biológicas, Consejo Superior de Investigaciones Científicas, Madrid, Spain; 3 CIBER de Enfermedades Raras (CIBERER), Madrid, Spain; 4 Departamento de Biología Molecular, Centro de Biología Molecular Severo Ochoa, Consejo Superior de Investigaciones Científicas-Universidad Autónoma de Madrid (CSIC-UAM), Madrid, Spain; 5 Unidad de Bioestadística, Instituto de Investigación Sanitaria La Fe, Valencia, Spain; 6 Servicio de Oftalmología, Hospital de Manises, Valencia, Spain; 7 Unidad de Genética, Hospital Universitario y Politécnico La Fe, Valencia, Spain

## Abstract

Growing evidence suggests that inflammation is involved in the progression of retinitis pigmentosa (RP) both in patients and in animal models. The aim of this study was to investigate the effect of Adalimumab, a monoclonal anti-TNFα antibody, on retinal degeneration in a murine model of human autosomal recessive RP, the *rd10* mice at postnatal day (P) 18. In our housing conditions, *rd10* retinas were seriously damaged at P18. Adalimumab reduced photoreceptor cell death, as determined by scoring the number of TUNEL-positive cells. In addition, nuclear poly (ADP) ribose (PAR) content, an indirect measure of PAR polymerase (PARP) activity, was also reduced after treatment. The blockade of TNFα ameliorated reactive gliosis, as visualized by decreased GFAP and IBA1 immunolabelling (Müller cell and microglial markers, respectively) and decreased up-regulation of TNFα gene expression. Adalimumab also improved antioxidant response by restoring total antioxidant capacity and superoxide dismutase activity. Finally, we observed that Adalimumab normalized energetic and metabolic pattern in *rd10* mouse retinas. Our study suggests that the TNFα blockade could be a successful therapeutic approach to increase photoreceptor survival during the progression of RP. Further studies are needed to characterize its effect along the progression of the disease.

Retinitis pigmentosa (RP) is a group of inherited retinal dystrophies characterized by progressive and irreversible loss of vision that in most models studied, parallels photoreceptor cell death^1^,^2^,^3^. RP is the leading cause of genetic blindness in adults with an estimated incidence of 1 in 3,500 ~ 4,500 human births^4^. Patients with RP typically loose night vision in adolescence, peripheral vision in young adulthood, and central vision later in life, due to progressive, sequential loss of rod and cone photoreceptor cells. Although many therapeutic approaches have been developed to prevent photoreceptor cell death, no effective treatment is still available. More than 70 genes, including phosphodiesterase 6 (PDE6) subunit genes, have been identified to date whose mutations cause different forms of RP^5^,^6^. Besides the genetic defect, evidence suggests that oxidative stress and neuroinflammation contribute to its progression^7^,^8^,^9^. In particular, inflammatory processes including microglial activation and upregulation of inflammatory cytokines (TNFα, IL-6, IL-1β, etc.) and chemokines (MCP1, RANTES, etc.) have been described in patients as well as in animal models of RP^10^,^11^,^12^.

Tumor necrosis factor alpha (TNFα) is a pleiotropic cytokine essential for the induction and maintenance of the inflammatory immune responses. It is a well characterized mediator of cellular activities including proliferation, survival, differentiation, inflammation and cell death. In the retina, it is likely secreted from activated macrophages, astrocytes, microglia and Müller glial cells. TNFα can trigger several well-characterized death-promoting (caspase-dependent and caspase-independent cell death) and survival-promoting pathways, depending upon the predominating signalling pathway in the particular cell type^13^. In the eye, TNFα appears to have a role in the pathogenesis of inflammatory diseases such as uveitis^14^, as well as in retinal degenerations such as diabetic retinopathy, age-related macular degeneration and, recently, in RP^11^,^15^,^16^,^17^.

Several anti-TNFα agents (Infliximab, Adalimumab, Etarnecept, Golimumab and Certolizumab pegol) have been developed and approved for clinical use in inflammatory diseases such as rheumatoid arthritis, psoriasis and ankylosing spondylitis^18^. These anti-TNFα agents are antibodies against TNFα or TNFα receptor. In Ophthalmology, they are widely used as an alternative to traditional immunosuppressive treatments in non-infectious uveitis. More recently anti-TNFα agents have been used for retinal diseases such as neovascular age-related macular degeneration, diabetic macular edema and retinal vein occlusions^19^,^20^,^21^.

To further explore the *in vivo* potential benefits of blocking TNFα we adopted the *rd10* mouse, a model of human autosomal recessive RP. This mouse carries a mutation on the β subunit of the cGMP PDE6 gene (*Pde6β*) that produces retinal degeneration^2^.

In this study, we first analysed the early stages of retinal degeneration and the profile of TNFα expression in *rd10* mice under our housing conditions. We observed a peak of TNFα gene expression and photoreceptor cell death at postnatal day (P) 18. Therefore, we studied the effect of Adalimumab, a monoclonal antibody against TNFα, on the progression of the retinal degeneration at this age. We observed that Adalimumab prevented TNFα upregulation, reduced photoreceptor cell death, slowed microglial and Müller cell activation, improved antioxidant response and ameliorated the energetic and metabolic dysfunction at P18. Based on these results we suggest that anti-TNFα therapies could be promising treatments to improve photoreceptor cell survival in humans with RP. However, further studies are needed to investigate the molecular mechanisms involved in the protective effect of anti-TNFα agents and their long-term effect.

## Results

### Temporal progression of retinal degeneration in *rd10* mice

**Figure 1:**
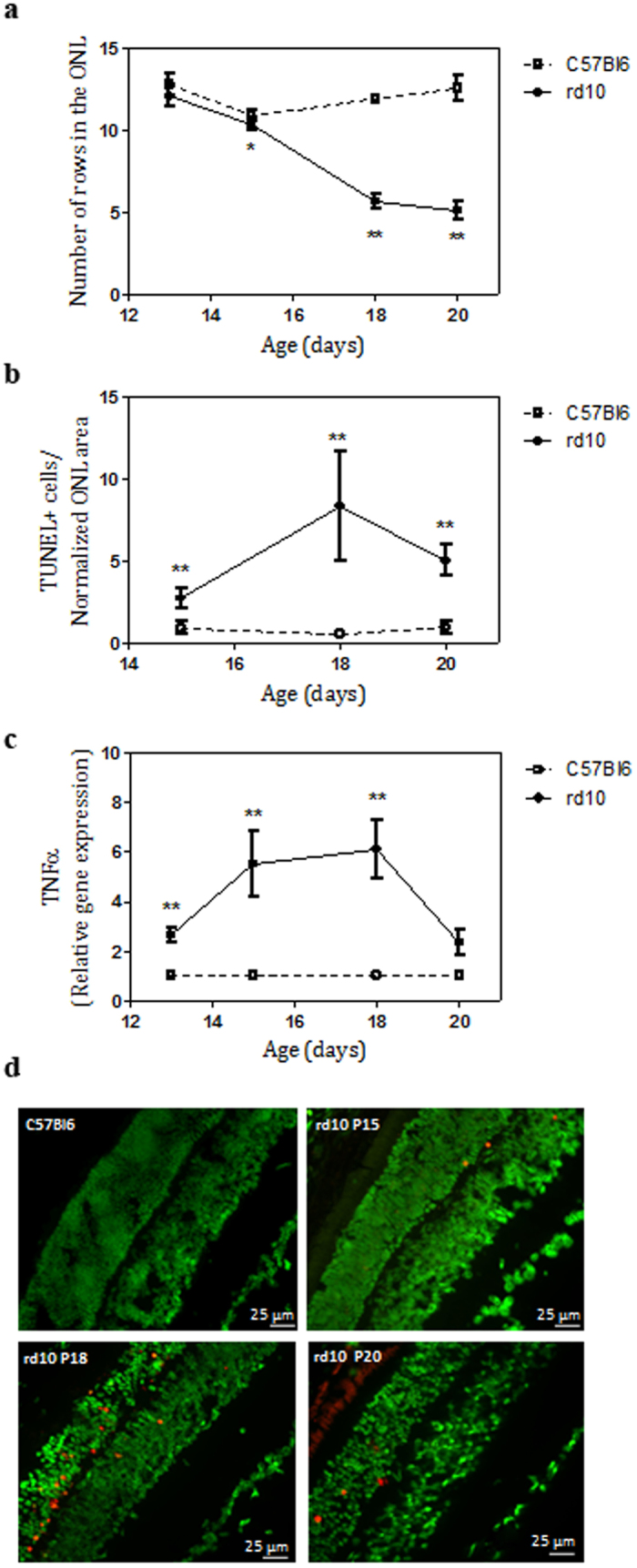
Time course of retinal degeneration in the *rd10* mouse retina. (**a**) Quantitation of the number of rows of photoreceptor nuclei from P13 to P20 in control (C57Bl6) and *rd10* mice. (**b**) Number of TUNEL-positive cells by normalized ONL thickness in control and *rd10* mice from P15 to P20. (**c**) Relative TNFα gene expression from P13 to P20 in *rd10* mice. Values are the mean ± SEM of, at least, five retinas per group. Values that are significantly different are indicated by asterisks *p < 0.05, **p < 0.001, ***p < 0.0001 (Mann-Whitney U test). (**d**) Photoreceptor cell death was visualized by TUNEL (red) in Sytox Green-counterstained in retinal sections from control mice at P18 and from *rd10* mice at P15, P18 and P20.

Light exposure accelerates progression of retinal degeneration in many animal models of RP^22^,^23^. Hence we first investigated the time course of retinal degeneration in *rd10* mice from P13 to P20 stabulated under light intensities between 98 and 131 lux. Sytox Green nuclear staining showed a decline of the number of photoreceptor cell nuclei rows in the outer nuclear layer (ONL) in *rd10* mice. As expected, we did not find a significant difference between control and *rd10* mice at P13. But the number of cell nuclei rows in the ONL dropped abruptly from eleven to five from P15 to P20 (Fig. 1A). Degeneration was evident at P18 (Fig. 1D).

Then, we assessed photoreceptor cell death by TUNEL staining which detects DNA breaks in both apoptotic and necrotic nuclei. From P15 to P20 there was a significant increase of TUNEL-positive cells in *rd10* mice, reaching a peak at P18 (12.5 ± 2.4 TUNEL-positive cells/normalized ONL area (five-fold increase between P15 and P18), Mann-Whitney U test, p < 0.0001) (Fig. 1B,D, and Supplementary Table S1).

It has been previously described that TNFα is upregulated in the retina of *rd10* mice previous to photoreceptor cell death peak^11^,^24^. Therefore, we analysed gene expression of TNFα during the time course of retinal degeneration in our housing conditions. As shown in Fig. 1C, TNFα was upregulated from P13 to P20 with the highest expression at P18 (6.1 ± 1.2 relative expression (six-fold increase over control retinas), Mann-Whitney U test, p = 0.0002) concomitantly with the peak of TUNEL-positive cells. At P18 a significant increase of TNFα protein level was also observed in the retina of *rd10* mice (mean = 65.5 pg/mg protein, confidence interval [25.1–170.9], generalized least squares model, p = 0.0034) compared to control retinas (mean = 17.1 pg/mg protein, confidence interval [15.8–18.5]).

In view of these results, P18 was chosen as the end point for the ensuing analysis of the potential protective effect of Adalimumab.

### Adalimumab reduced photoreceptor cell loss and PARP activation

**Figure 2:**
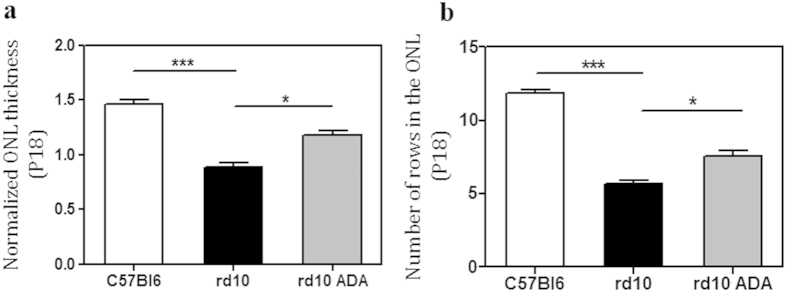
Adalimumab administration decreased photoreceptor cell loss in the *rd10* mouse retina at P18. Bar graph illustrates the effect of Adalimumab (ADA) on normalized ONL thickness (**a**) and number of rows of nuclei in the ONL (**b**) Values are the mean ± SEM of six retinas per group. Values that are significantly different are indicated by asterisks *p < 0.05, ***p < 0.001 (Mixed linear model analysis).

To determine whether Adalimumab treatment protects against photoreceptor cell death, we quantified the normalized ONL thickness ratio and the number of rows of nuclei in the ONL at P18. Normalized ONL thickness decreased slower in Adalimumab-treated *rd10* retinas (reduction of 20% over control retinas) than in vehicle-treated *rd10* retinas (reduction of 40% over control retinas) (Fig. 2A and Supplementary Table S1). A mixed linear model analysis also revealed that Adalimumab significantly reduced (p = 0.047) photoreceptor cell loss (7.6 ± 0.4 number of rows of nuclei in the ONL) compared to vehicle-treated *rd10* retinas (5.9 ± 0.3 number of rows of nuclei in the ONL) (Fig. 2B and Supplementary Table S1).

**Figure 3:**
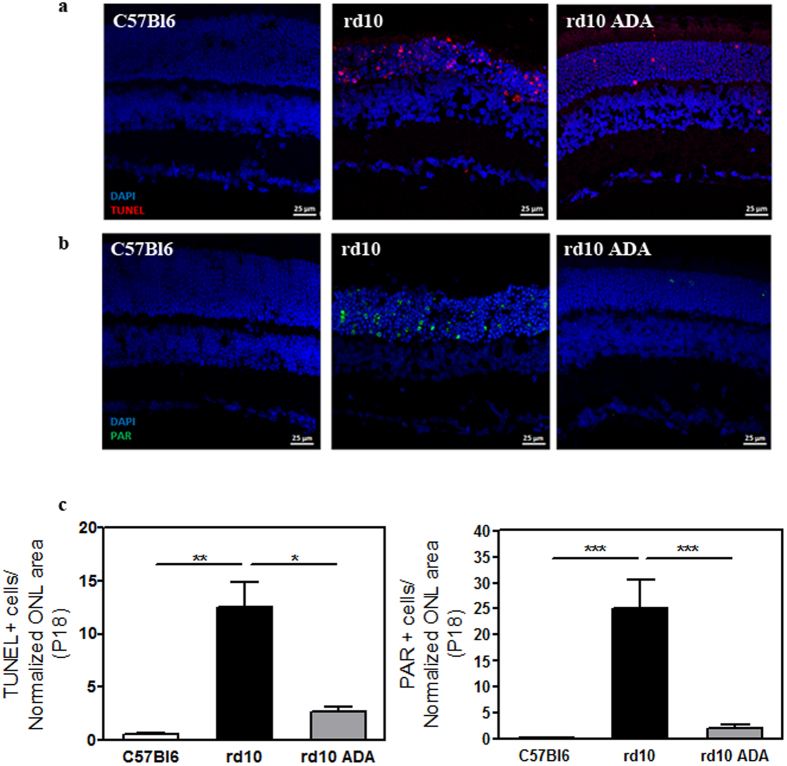
Adalimumab significantly reduced photoreceptor cell death in the *rd10* mouse retina at P18. Representative photomicrographs of retinal sections showing (**a**) TUNEL-stained sections revealing dead photoreceptors in DAPI-counterstained and (**b**) PAR content in DAPI-counterstained retinal sections. (**c**) Bar graph illustrates the effect of Adalimumab (ADA) on the number of TUNEL-positive nuclei and PAR-positive cells. Values are the mean ± SEM of, at least, five retinas in each group. Values that are significantly different are indicated by asterisks *p < 0.05, **p < 0.01, ***p < 0.001 (Mixed linear model analysis).

We previously described that Infliximab, other monoclonal anti-TNFα antibody, significantly reduced the number of TUNEL-positive cells in Zaprinast-treated explants of porcine retina^25^. In the current study we observed that intraperitoneal administration of Adalimumab significantly decreased (∼78%, p = 0.048) the number of TUNEL-positive cells in the ONL at P18 (2.7 ± 0.4 TUNEL-positive cells) compared to vehicle-treated *rd10* retinas (12.5 ± 2.4 TUNEL-positive cells) (Fig. 3A,C, and Supplementary Table S1). Therefore the blockade of TNFα was preventing photoreceptor cell loss in *rd10* mice. However, the mechanisms of TNFα-induced cell death remained unclear.

Paquet-Durand *et al.* (2007) demonstrated non-apoptotic mechanisms involved in photoreceptors cell death such as the activation of PARP which led to necroptosis in a similar mouse model of RP^26^. To assess whether TNFα inhibition prevented PARP activation, we visualized the content of PAR polymers (an indirect marker for PARP activity) and its retinal location at P18. As shown in Fig. 3B, an intense and nuclear PAR staining was found in photoreceptor cells of vehicle-treated *rd10* retinas. In contrast, Adalimumab substantially decreased (up to 90%, p < 0.001) nuclear PAR staining in photoreceptor cells (2.1 ± 0.6 PAR-positive cells), compared to vehicle-treated *rd10* retinas (25.1 ± 5.5 PAR-positive cells) (Fig. 3C). Taking together these results suggested that Adalimumab could slow down photoreceptor cell death through PARP pathway inhibition in *rd10* mice. Further studies will be designed to test this hypothesis.

### Adalimumab reduced reactive gliosis

**Figure 4:**
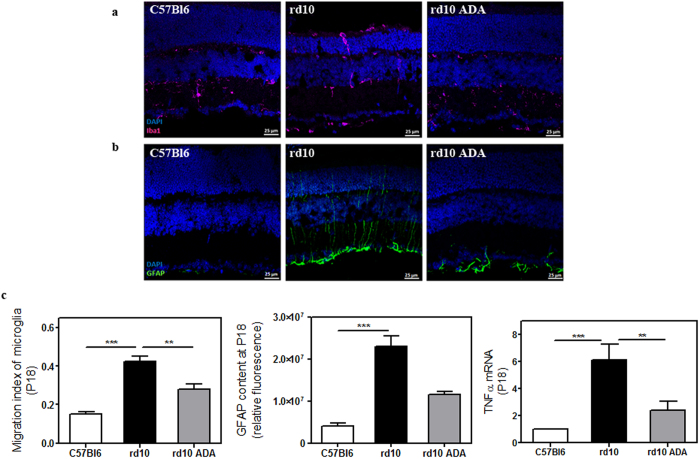
Adalimumab ameliorated reactive gliosis in the *rd10* mouse retina at P18. Representative photomicrographs of retinal sections showing (**a**) Iba1-labelling to visualize microglial cells and (**b**) GFAP content in DAPI-counterstained sections. (**c**) Bar graph illustrates the effect of Adalimumab (ADA) on migration index of microglia, the corrected fluorescence of GFAP content and TNFα gene expression. Values are the mean ± SEM of, at least, five retinas per group. Values that are significantly different are indicated by asterisks **p < 0.01, ***p < 0.001 (Mixed linear model analysis).

Chronic microglia activation is associated with various neurodegenerative diseases including RP. We investigated whether Adalimumab treatment reduced microglial activation in *rd10* mouse retinas. Microglial cells were identified by specific labelling with Iba1, a microglia/macrophage-specific calcium-binding protein. The level of migration from the inner to the outer layers of retina was employed as an indicator of microglial activation. Iba1 immunostaining revealed that microglia was located in all layers, especially in outer layers in vehicle-treated *rd10* retinas. Conversely, after intraperitoneal administration of Adalimumab, microglia was preferentially located in the inner layers of the retina. A few microglial cells were observed in the outer plexiform layer but not in the ONL in Adalimumab-treated *rd10* retinas (Fig. 4A). To quantify microglial behaviour, we performed a weighted analysis where values close to 1 indicate a high rate of microglial migration to outer layers. Statistical analysis confirmed that microglial activation was reduced (p = 0.006) in Adalimumab-treated *rd10* retinas (0.28 ± 0.03) compared to vehicle-treated *rd10* retinas (0.43 ± 0.02) at P18 (Fig. 4C and Supplementary Table S1).

Gliosis commonly involves upregulation of the glial fibrillary acidic protein (GFAP), an intermediate filament protein, in Müller glial cells. We also studied whether the blockade of TNFα with Adalimumab ameliorates glial activation in *rd10* mice. Figure 4B shows that GFAP was upregulated in vehicle-treated *rd10* retinas (p < 0.001) compared to control retinas. Adalimumab tended to decrease (up to 50%) (p = 0.27) GFAP-positive immunolabelling compared to vehicle-treated *rd10* retinas at P18 (Fig. 4C).

After evaluating the effect of Adalimumab in prevention of reactive gliosis, we analysed whether Adalimumab affected gene expression of the inflammatory mediators TNFα and IL-6 by real time PCR at P18. After Adalimumab treatment, TNFα expression was significantly reduced (p = 0.006) (Fig. 4C and Supplementary Table S1). At P18 we did not observe changes in IL-6 expression neither in vehicle-treated *rd10* retinas nor in Adalimumab-treated *rd10* retinas (data not shown).

### Adalimumab ameliorated energetic and metabolic dysfunction

It has been described a coordinated downregulation of several metabolic pathways at the onset of retinal degeneration, affecting amino acid biosynthesis, glycerol phospholipid metabolism and oxidative phosphorylation in 14-d-old *Rh1P37H* retinas, a *Drosophila* model expressing the equivalent of the most common RP-linked mutation, *RhoP23H*^27^. First, we assessed whether *rd10* mice presented a deregulated expression of enzymes of energy metabolism and mitochondrial structure and dynamics at P18 by quantitative reverse phase protein microarrays (RPPM)^28^ using validated antibodies (Supplementary Figure S1). We analysed the expression of proteins of glycolysis (GAPDH, LDHA), electron transport and oxidative phosphorylation (OXPHOS) (NDUFS3, SDHB, CORE 2, COXII and β-F1-ATPase), mitochondrial structure (HSP60) and dynamics (OPA1), β-oxidation (HADHA), the pentose phosphate pathway (G6PDH), the antioxidant response (SOD1, SOD2 and catalase) and the expression of β-actin as reference protein (Supplementary Figure S1).

At the onset of RP, the expression of most of these markers was significantly downregulated in retinas of *rd10* mice leading to a global energy depletion that might contribute to cell death (Table 1). No significant differences in β-actin were found between control (0.64 ± 0.05 arbitrary units (a.u.)/ng total protein) and *rd10* retinas (0.71 ± 0.06 a.u./ng total protein). Hence we observed an energy depletion and mitochondrial dysfunction during photoreceptor cell death. As shown in Table 1, intraperitoneal administration of Adalimumab significantly ameliorated this phenotype in retinas from *rd10* mice at P18.

### Adalimumab improved antioxidant response

It has been reported that inflammatory processes and oxidative stress are closely linked in several pathologies. The contribution of oxidative damage to cell death in *rd10* mouse retinas has been previously described^29^,^30^,^31^. In particular, anti-TNFα treatments have been demonstrated to have a positive effect on improving antioxidant response and reducing the production of reactive oxygen species (ROS) in several pathological situations^32^. Anti-TNFα antibodies decrease cytokine concentrations, thus directly influencing ROS/RNS production by inflammatory cells or indirectly modulate ROS-stimulated signalling pathways activity. Therefore we evaluated whether Adalimumab improved the antioxidant response in retinas from *rd10* mice. For this purpose, we measured the total antioxidant capacity (TAC), enzymatic activity of superoxide dismutase (SOD) and catalase (CAT) which play a major role in the first line of antioxidant defence.

**Figure 5:**
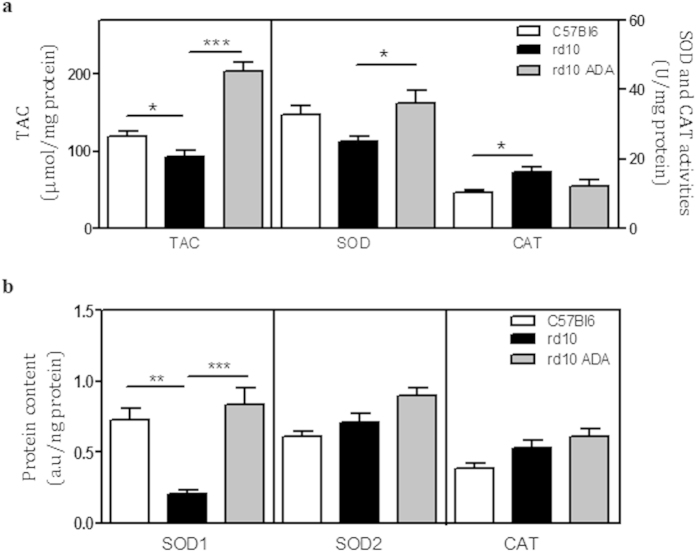
Adalimumab improved antioxidant response at P18. Total antioxidant capacity (TAC), superoxide dismutase (SOD) and catalase (CAT) activities (**a**) and protein content of SOD1, SOD2 and CAT (**b**) were measured. Quantitation is shown in bar graphs. Values are the mean ± SEM of, at least, six retinas per group. Values that are significantly different are indicated by asterisks *p < 0.05, **p < 0.01, ***p < 0.001 (Mixed linear model analysis).

The TAC was reduced (p = 0.031) in vehicle-treated *rd10* retinas (92.6 ± 7.8 nmol of Trolox/mg protein) compared to control retinas (119 ± 5.7 nmol of Trolox/mg protein). Adalimumab significantly increased TAC to 204.5 ± 11.6 nmol of Trolox/mg protein (p < 0.001) (Fig. 5A and Supplementary Table S1).

Total SOD activity seemed to decrease (p = 0.12) in vehicle-treated *rd10* retinas (25 ± 1 U/mg protein) compared to control retinas (33 ± 2 U/mg protein). However, Adalimumab-treated *rd10* retinas showed higher values of total SOD activity (p = 0.042), being similar to control retinas (36 ± 4 U/mg protein) (Fig. 5A and Supplementary Table S1).

Analysis of the protein content of the cytosolic (SOD1) and mitochondrial (SOD2) antioxidant enzymes superoxide dismutase and CAT by RPPM suggested that the diminished TAC and SOD activities could be due to a very large reduction of the expression of SOD1 in vehicle-treated *rd10* retinas (0.20 ± 0.03 a.u/ng protein, p = 0.004) (Fig. 5B). The content of SOD2 in vehicle-treated *rd10* retinas (0.71 ± 0.06 a.u/ng protein) was similar to control retinas (0.61 ± 0.04 a.u/ng protein). Adalimumab increased the content of SOD1 (0.84 ± 0.11 a.u/ng protein, p = 0.001) and, to a lesser extent, of SOD2 (0.90 ± 0.05 a.u/ng protein, p = 0.087) (Fig. 5B and Supplementary Table S1).

CAT activity was higher (p = 0.011) in vehicle-treated *rd10* retinas (16 ± 2 U/mg protein) than in control retinas (10 ± 1 U/mg protein). CAT content had a tendency to increase (0.53 ± 0.05 a.u/ng protein, p = 0.15) compared to control retinas (0.39 ± 0.03 a.u/ng protein). Adalimumab had a tendency to reduce CAT activity (p = 0.078) but it did not have effect on CAT content (Fig. 5A,B, and Supplementary Table S1).

## Discussion

TNFα inhibitors have been widely used for the treatment of different autoimmune disease, including ocular inflammatory disorders, with promising results^33^,^34^,^35^. Nevertheless, to our knowledge, we present the first study that evaluates the effect of TNFα inhibitors on the progression of the retinal degeneration in a murine model of RP. The results obtained in the present study revealed that intraperitoneal administration of Adalimumab, a recombinant human monoclonal antibody against TNFα, reduced retinal degeneration decreasing photoreceptor cell death and reactive gliosis at early stages of RP in *rd10* mice.

We used the *rd10* mouse model of human autosomal recessive RP. This mutant mouse mimics the disease progression seen in some human conditions and offers an amenable therapeutic window. In general, rod photoreceptors start to degenerate between P16 and P20, with a maximum cell death around P21 and P25. By P60, rods are undetectable and only cones remain^2^,^36^. Under our housing conditions, we observed that photoreceptor degeneration started around P15, with maximum TUNEL-positive cells around P18. This time point of maximum cell death agreed with results recently described by Arango-Gonzalez *et al.* (2014)^37^. In our study light intensity was higher (98–131 lux) than light intensity described by other authors (around 60 lux)^38^,^39^. Therefore, the early onset of photoreceptor degeneration could be explained by light conditions.

In the present study, we demonstrated that intraperitoneal injections of Adalimumab displayed a neuroprotective effect on photoreceptors survival at the onset of their death in *rd10* mice. The administration of 3 mg/kg Adalimumab from P9 to P17 contributed to preserve retinal structure reducing the loss of photoreceptors at P18. Further, Adalimumab strongly reduced the number of TUNEL-positive cells in the ONL. Based on several studies using different animal models of RP, it has been suggested that apoptosis is playing a minor role in retinal degeneration^37^. However, non-apoptotic mechanisms seem to prevail as major contributors to cell death including accumulation of cGMP and activation of histone deacetylase (HDAC), PARP (PAR accumulation) and calpains^40^,^41^.

On the other hand, TNFα is considered both apoptotic and necroptotic inducer mainly through its binding to the receptor TNFR1. TNFα binds to TNFR1 leading to the recruitment of multiple proteins that can trigger different downstream pathways as activation of the transcription factor NF-κB, MAPKs (mitogen-activated protein kinases), caspase 8 promoting cell survival, apoptosis or necroptosis, etc^42^. The two most extensively studied ways of necroptosis are initiated by TNFR1 and by PARP pathway. PARP pathway is initiated by overactivation of the DNA repair enzyme PARP-1, resulting in the massive synthesis of PAR and, eventually in necroptosis^43^. An evident controversial exists concerning to the link between TNFα and PARP pathway during necroptosis. Previously, PARP pathway has been suggested as a key element of TNFα-mediated necroptosis^44^. But other authors propose that TNFα-induced necroptosis and PARP pathway represent distinct and independent routes to necroptosis^45^,^46^. In our previous study we found that TNFα inhibition did not prevent PAR accumulation but also increased it supporting the idea that PARP pathway was independent of TNFα-associated pathways in cultures of porcine retina exposed to Zaprinast^25^. In the current study we confirmed that there was an excessive nuclear PAR content in vehicle-treated *rd10* mouse retinas that was effectively prevented by Adalimumab treatment. Therefore our results suggest that PARP activation is involved in TNFα-mediated photoreceptor cell death in *rd10* mice. As described above, PARP activation could be consequence of excessive cGMP accumulation that in turns would activate HDAC. PARP activation could be also consequent of the inflammatory process. We should confirm whether reduced inflammation could be the primary responsible for down regulation of PARP activity or could be secondary to reduced cell death. Thus, the mechanistic interpretation of these results is complicated and deserves further studies.

Upregulation of TNFα and activation of microglial cells are early events involved in photoreceptor death in murine models of RP^11^,^47^. We corroborated microglial activation and migration to outer retina in *rd10* mice. However, we did not visualize Iba1-positive cells in the ONL in retinas from *rd10* mice treated with Adalimumab. Thus, inhibition of TNFα reduced excessive microglial activation and migration of microglial cells to the outer layers. Microglial activation is a hallmark of neuroinflammation. It is associated with various neurodegenerative diseases including retinal degenerations^10^,^48^. Some therapeutic approaches are designed to inhibit microglial activation. For instance, minocycline attenuated photoreceptor cell death in different animal models of retinal degeneration^49^,^50^ and improved vision in patients with diabetic macular edema^51^. Around 80 clinical trials are in progress to investigate the effect of minocycline in several pathologies including retinal degenerations (ClinicalTrials.gov, Identifiers: NCT02140164 and NCT01468844).

Recent studies showed that Adalimumab decreased glial cell activation (overexpression of GFAP) and preserved retinal organization in organotypic cultures of porcine neuroretina exposed to TNFα^52^. Activated Müller cells can release antioxidants, growth factors and cytokines, including TNFα, contributing to retinal regeneration or degeneration. Müller cells are activated in models of RP resulting in overexpression of GFAP, activation of ERK (extracellular signal-regulated kinase), translocation of Müller cell bodies to the outer retina and thickening of their processes^25^,^53^. In the present study, Adalimumab tended to ameliorate GFAP overexpression in *rd10* retinas.

Adalimumab specifically blocks the interaction of soluble TNFα, and to a lesser extent, transmembrane TNFα with its receptors, inhibiting downstream cellular signalling mechanisms^21^. We found that blocking TNFα tends to normalize the increased TNFα production, as described in other pathological situations^54^. TNFα blockade prevented the activation of signalling pathways involving the activation of p38 MAPK or NF-κβ^55^,^56^. MAPKs such as ERK, JNK (c-Jun N-terminal kinase) and p38, are involved in many inflammatory and degenerative processes including retinal cell death^53^. Thus, we speculate that Adalimumab could attenuate the inflammatory process by interfering downstream signalling of TNFα, at the level of p38 MAPK or ERK activation. Further studies are needed to confirm this hypothesis.

Inflammation and oxidative stress are closely linked processes in several pathological situations. It has been widely suggested that oxidative stress contributes to the pathogenesis of RP in animal models and patients^29^,^30^,^31^. For instance, we previously found that RP patients present a reduced antioxidant response^9^. In the current study we assessed whether down regulation of inflammation (TNFα and reactive gliosis) induced by Adalimumab also influenced antioxidant response. Adalimumab improved antioxidant defence machinery in *rd10* mouse retinas. This treatment improved the total antioxidant capacity and total SOD activity probably by increasing SOD1 content in the retina of the *rd10* mouse. It has been shown that SOD1 deficient mice are more sensitive to the damaging effects derived of oxidative insults^57^,^58^. Thus, SOD1 seems to be a key defender against oxidative damage in retina. It has been previously shown that Adalimumab can influence the antioxidant and oxidant status in other pathological situations such as psoriasis, rheumatoid arthritis, ankylosing spondylitis or uveitis^32^,^59^,^60^. For instance, Adalimumab is capable of upregulating SOD activity and downregulating catalase activity in mesenchymal stem cells from skin of patients with psoriasis^59^ and to restore glutathione (GSH) content and glutathione peroxidase activity in eyes of a uveitis experimental model^60^.

The retina is an organ with one of the highest energy demands per tissue weight due to the photoreceptor cell metabolism. Glucose is metabolized through two major metabolic pathways: (1) glycolysis to obtain energy (ATP), reducing power (NADH) and pyruvate and (2) the pentose phosphate pathway (PPP) to obtain reducing power (NADPH) and ribose for nucleic acid biosynthesis. Under aerobic conditions, pyruvate supplies ATP, precursors of certain amino acids as well as NADH through the citric acid cycle or Krebs cycle (TCA cycle). The NADH generated by the TCA cycle is fed into the mitochondrial oxidative phosphorylation (OXPHOS) pathway. During OXPHOS, electrons are transferred from electron donors to electron acceptors such as oxygen in redox reactions releasing energy and finally ATP. Photoreceptors contain a dense concentration of mitochondria in their inner segments that provides the ATP for the phototransduction processes mainly through the OXPHOS pathway^61^,^62^. Part of the oxygen consumed in OXPHOS is transformed to reactive oxygen species which are converted to H_2_O by antioxidant enzymes. However, a deficient antioxidant defence results in oxidative damage that initiates a cascade of events resulting in mitochondrial dysfunction and cell death^63^. In this study we corroborated the downregulation of OXPHOS pathway observed at the onset of photoreceptor degeneration in a *Drosophila* model expressing the equivalent of the most common RP-linked mutation, *RhoP23H*^27^. We also observed significant alterations in other energetic pathways as glycolysis, pentose phosphate pathway and β-oxidation indicating global energy depletion in *rd10* retinas at P18. We also detected mitochondrial alterations in proteins related to its dynamics and structure supporting the results reported by other authors^63^,^64^. Adalimumab ameliorated energetic and metabolic dysfunction in *rd10* mice.

It is tempting to speculate that Adalimumab or other anti-TNFα agents could be a promising therapy for RP and other retinal degenerations. However, further studies are needed to investigate in detail the molecular mechanisms involved in the neuroprotective effect of Adalimumab and its long-term effect.

## Methods

### Animals and treatment

*rd10* mice were used as human model of autosomal recessive retinitis pigmentosa. Wild-type C57Bl6 mice with the same genetic background as *rd10* mice were used as control. Mice were kept under a 12 hours light/dark cycle, humidity and temperature controlled and with food and water supplied *ad libitum*. All cages were placed on the lower shelf of an IVC rack with light illuminance of 115 ± 7 lux (95% CI: 98–131). Mice were housed in the Animal Facility of *Unitat Central d’Investigació* (UCIM) of Valencia University. This study was carried out in accordance with guidelines from the European Union Guidelines for the Care (European Union Directive (2010/63/EU) and the Use of Laboratory Animals. All animal procedures and protocols were approved (A1361179906177) and monitored by the Committee of Ethics in Research of the Faculty of Medicine, University of Valencia.

To determine the profile of retinal degeneration at early stages in our housing conditions, untreated *rd10* and C57Bl6 mice were euthanized at P13, P15, P18 and P20. The eyes were rapidly removed and processed as described below.

To evaluate the effect of Adalimumab, each *rd10* mouse received one intraperitoneal injection of Adalimumab (Humira, Abbot Laboratories) saline solution at 3 mg/kg every three days starting at P9 and until P17. The dose of 3 mg/kg of Adalimumab was chosen based on previous studies with murine models of rheumatoid arthritis and uveitis^60^,^65^,^66^. No apparent side effects (e.g. opportunistic infections) were detected in animals treated with Adalimumab.

Control mice received the same volume of saline (vehicle) at the same time. At P18, treated and untreated *rd10* mice and C57Bl6 mice were sacrificed. The eyes were rapidly removed and processed as described below.

### ELISA assay

Retinas were homogenized in 20 mM Tris-HCl pH 7.4, 10 mM EDTA containing protease inhibitor cocktail (Complete Protease Inhibitor Cocktail; Roche, Basel, Switzerland) and 200 μM phenylmethylsufloxifluoride (PMSF). The TNFα protein levels were estimated with a high sensitivity ELISA kit (eBioscience, Ireland, UK), according to the manufacturers’ instructions. Tissue TNFα levels were expressed as pg/mg protein.

### Tissue processing

To obtain retinal sections, the eyes were rapidly removed and fixed in 4% filtered paraformaldehyde for two hours at room temperature and cryoprotected in a sucrose gradient (15–20–30%). Eyes were frozen embedded in OCT and 10 μm sections were cut in a cryostat (Leica CM1900, Nussloch, Germany).

For biochemical determinations and gene expression analysis, retinas were isolated, placed immediately into the appropriate buffer and stored at −80 °C.

### Retinal immunohistochemistry and TUNEL assay

Immunofluorescent staining procedures were performed in 10 μm cryosections. Sections were post-fixed in 4% filtered paraformaldehyde (Sigma-Aldrich, Madrid, Spain) in 0.1 M phosphate buffer pH 7.4 for 15 minutes at room temperature. Sections were pre-treated with citrate buffer pH 6.0 for epitope retrieval and incubated in blocking solution containing 5% normal goat serum, 1% bovine serum albumin and 0.25% Triton X-100 for one hour. Afterwards, they were incubated with primary antibody anti-PAR (1:200, Enzo Life Science, Madrid, Spain) as an indirect marker for PARP activity, anti-Iba1 (1:300, Wako Pure Chemical Industries Ltd., Osaka, Japan) and anti-GFAP (1:400, Sigma-Aldrich, Madrid, Spain) overnight at 4 °C. Sections were then incubated with the fluorescence-conjugated secondary antibody Alexa Fluor 647, 555 or 488 (Invitrogen, Life Technologies, Madrid, Spain) for one hour at room temperature. After labelling, the sections were mounted in Fluoromount-G (Southern Biotechnology, Birmingham, AL, USA) and observed under a fluorescence or confocal microscope.

To evaluate cell death, the terminal deoxynucleotidyl transferase dUTP nick and labelling (TUNEL) assay was used as previously described^67^.

### Microscopy and quantification

The retinal sections were examined under an Eclipse 80i microscope (NIKON Instruments, Badhoevedorp, The Netherlands) or under a confocal microscope (Leica TCS SP5 Confocal microscope, Leica Microsistemas SLU, Barcelona, Spain) belonging to the Microscopy Unit of the IIS-La Fe (Valencia, Spain). ImageJ software was used to quantify the thickness and the number of rows of photoreceptor nuclei in the ONL, the number of TUNEL, PAR and Iba1-positive cells and the corrected fluorescence of GFAP.

We measured the ONL thickness of the entire retina normalized to the thickness of the inner nuclear layer (INL) to avoid the bias derived of the angle of the sectioning plane. Because the ONL thickness and the degenerative process in the *rd10* model are not uniform and dependent upon location, we performed several measurements across the entire retina (from the nasal to the temporal retina) for each mouse. The *normalized ONL thickness ratio* is defined as the ONL thickness/INL thickness. At least five entire retinas were analysed per experimental group.

The number of TUNEL or PAR-positive cells was represented as the ratio between the number of TUNEL or PAR-positive cells in the ONL and the normalized ONL thickness ratio of each section.

To evaluate microglial activation we measured the migration index (M.I) which is defined as the number of Iba1-positive cells weighted according to the retinal layer where are located (M.I = ∑(number of Iba1-positive cells in each layer × layer weighted factor)/total number of Iba1-positive cells in the section. The layer weighted factor was 1 for ONL, 0.5 for outer plexiform layer (OPL) and 0.25 for INL.

Corrected fluorescence of GFAP was quantified as previously described^25^. TUNEL and PAR-positive cells, microglial migration index and the corrected fluorescence of GFAP were quantified from four non-adjacent sections of at least five retinas for each experimental group.

### Isolation of total RNA and cDNA synthesis

Total RNA was isolated from frozen retina using RNeasy mini kit (Qiagen, Hilden, Germany) following the manufacturer’s protocol. Then, cDNA was synthesized starting from 1 μg of RNA by reverse transcription using the GeneAmp Gold RNA PCR Reagent kit (Applied Biosystems, Carlsbad, CA, USA) following manufacturer’s instructions.

### Quantitative real time PCR

The relative expression of TNFα and IL-6 was measured by real-time PCR using a commercial thermal-cycler (Applied Biosystems ViiA^TM^ 7 Real-Time PCR System; Life Technologies Corporation, Carlsbad, California, USA), TaqMan® gene expression assay (Mm00443260_g1 (TNFα) and Mm00446190_m1 (IL-6) and TaqMan® 2X PCR Master Mix (Applied Biosystems, Life Technologies Corporation, Carlsbad, California, USA). β2 microglobulin (β2m) gene (Mm00437762_m1) was used as housekeeping gene.

Real-time PCR was performed with 1 cycle of 2 min at 50 °C, followed by 1 cycle of denaturation of 10 min at 95 °C, continued by 40 cycles of 15 seconds denaturation at 95 °C and 60 seconds annealing at 60 °C.

### Evaluation of antioxidant response

Retinas were homogenized in 5 mM phosphate buffer pH 7, 0.9% NaCl, 0.1% glucose, centrifuged at 10,000 × g (total antioxidant capacity (TAC) and catalase (CAT) activity) or 1,500 × g (superoxide dismutase (SOD) activity) for 15 minutes at 4 °C. Supernatants were used to determine TAC, CAT and SOD activities with commercial kits following Manufacturer´s Instructions (Cayman Chemical, Ann Arbor, MI, USA). Protein concentrations were measured by the bicinchoninic acid (BCA) protein assay. For total SOD activity assay one unit of SOD was defined as the amount of enzyme needed to exhibit 50% dismutation of the superoxide radical. For CAT activity assay one unit was defined as the amount of enzyme that will cause the formation of 1.0 nmol of formaldehyde per minute at 25 °C. SOD and CAT activities were expressed as U/mg protein. Retinal TAC levels were expressed as nmol/mg protein.

Retinal content of SOD1 (cytosolic), SOD2 (mitochondrial) and CAT enzymes were measured as described below^28^.

### Reverse Phase Protein Microarrays (RPPM)

Thirty-six retinas were prepared for printing onto RPPM. Frozen retinas were grinded with liquid nitrogen and the tissue powder was extracted with 90 μl of 50 mM Tris/HCl pH 8, containing 100 mM NaCl, 1 mM DTT, 1% (v/v) Triton X100, 0.1% SDS, 0.4 mM EDTA and a cocktail of protease (Roche) and phosphatase (Sigma) inhibitors. After protein extraction, samples were centrifuged at 15,000 g for 30 min at 4 °C. The protein concentration in the supernatants was determined with the Bradford reagent (Bio-Rad Protein Assay) using BSA as standard. For printing and processing, samples were diluted in PBS to a final protein concentration of 0.75 μg/μl. Serially diluted protein extracts (0–1 μg/μl) derived from HCT-116 and OVCAR8 carcinoma cells were also prepared to assess printing quality and the linear response of protein recognition by the antibodies used^28^. A solution of BSA (1 μg/μl) was also prepared for printing as internal negative control. Approximately, 1 nl volume of each sample was spotted in duplicate onto nitrocellulose-coated glass slides (FAST Slides, Scheleicher & Schuell BioScience, Inc.) using a BioOdyssey Calligrapher MiniArrayer printer (Bio-Rad Laboratories, Inc.) equipped with a solid pin (MCP310S) at constant humidity and temperature^28^. After printing, arrays were allowed to dry and further blocked in PBS-T containing 5% skimmed milk. After, the arrays were incubated overnight at 4 °C with the indicated concentrations of the following highly specific primary monoclonal antibodies (mAbs): β-F1-ATPase (0,4 μg/ml) and anti-Hsp60 (4 ug/ml)^28^; anti-LDHA (4 μg/ml)^68^; anti-GAPDH (1/20000; Abcam), anti-complex II subunit B (SDHB, 1/500; Invitrogen), anti-complex III subunit Core 2 (Core2, 1/1000; Mitosciences), anti-catalase (1/5000; Sigma-Aldrich), anti-complex IV subunit II (COXII, 1/100; Abcam), anti-complex I Ndufs3 (1/200; Abcam), anti-β-actin (1/5000;Sigma), anti-OPA1 (1/500; BD Transduction Lab) and the indicated concentrations of the following polyclonal antibodies: anti-HADHA (1/200; Abcam), anti-SOD1 (1/200; Santa-Cruz), anti-SOD2 (1/200; Abcam) and anti-G6PDH (1/500; Thermo Scientific). After incubation, the arrays were processed and revealed as previously described^28^. The mean fluorescent intensity of the spots was quantified using FIJI software (N.I.H., USA) and converted into arbitrary units of expressed protein/ng of total protein in the tissue extract using the expression obtained in the linear plot of the HCT116 cell line as standard (see Supplementary Figure S1).

### Statistical analyses

Statistical analysis was performed using R software (version 3.1.2). Comparisons between different age categories were performed using Mann-Whitney U. A generalized least squares model was fitted to the log-transformed TNFα protein values to assess differences between the two experimental groups and also the effect of Adalimumab (ADA). Linear models were used to assess the association between ADA and the two experimental groups with the different response variables. To account for the non-independence of observations in the case of repeated measures per sample, a random intercept was added to the linear models with the variable sample as random factor. P values for the mixed linear models were computed using Satterthwaite approximation to degrees of freedom. Estimations for each variable in a linear mixed model are interpreted similarly than estimations from an ordinary linear model, that is, in an additive way. Negative values indicated a negative effect of being rd10 or having received Adalimumab. And positive values indicated a positive effect. Some response variables were logarithmically transformed to avoid negative values for the estimated control values. Result tables from the models are shown in Supplementary Table S1. Reverse Phase Protein Microarrays were analysed by One-way ANOVA and Newman-Keuls post-test. A p-value < 0.05 was considered statistically significant.

## Additional Information

**How to cite this article**: Martinez-Fernandez de la Camara, C. *et al.* Adalimumab Reduces Photoreceptor Cell Death in a Mouse Model of Retinal Degeneration. *Sci. Rep.* **5**, 11764; doi: 10.1038/srep11764 (2015).

## Supplementary Material

Supplementary Information

## Figures and Tables

**Table 1:** Summary of proteomic changes in *rd10* mice with or without Adalimumab.

	Protein	C	*rd10*	*rd10* ADA
OXPHOS	*NDUFS3*	3.1 ± 0.1	1.5 ± 0.2*	2.4 ± 0.2^a^
	*SDHB*	2.1 ± 0.1	1.3 ± 0.1*	2.1 ± 0.2^a^
	*CORE2*	1.4 ± 0.1	0.8 ± 0.1*	1.3 ± 0.1^a^
	*COX II*	2.0 ± 0.1	0.9 ± 0.1*	2.1 ± 0.2^a^
	*Β-F1*	1.6 ± 0.1	0.9 ± 0.1*	1.8 ± 0.1^a^
Glycolysis	*GADPH*	2.0 ± 0.2	1.2 ± 0.1*	2.1 ± 0.2^a^
	*LDHA*	0.70 ± 0.02	0.34 ± 0.04*	0.60 ± 0.05^a^
Pentose phosphate pathway	*G6PDH*	0.48 ± 0.04	0.36 ± 0.05*	0.64 ± 0.07^a^
β-oxidation	*HADHA*	0.34 ± 0.02	0.17 ± 0.01*	0.37 ± 0.03^a^
Mitochondrial dynamics	*OPA1*	1.9 ± 0.1	1.0 ± 0.1*	1.8 ± 0.2^a^
Mitochondrial structure	*HSP60*	0.13 ± 0.01	0.05 ± 0.01*	0.14 ± 0.01^a^

Note: Values are expressed as the mean ± SEM of fluorescence intensity (arbitrary units) per ng of total protein of six retinas per group. Values that are significantly different from C57Bl6 (C) are indicated by asterisks *p < 0.05. ‘a’ represent statistical differences (p < 0.05) between *rd10* and *rd10* ADA (One way ANOVA, Newman-Keuls post-test). ADA: Adalimumab.
