# Supplementary material for: Adalimumab Reduces Photoreceptor Cell Death in A Mouse Model of Retinal Degeneration
Source: Sci Rep. 2015 Jul 14;5:11764. doi: 10.1038/srep11764 (PMC4501000; doi:10.1038/srep11764)
Supplement: Supplementary Information [file srep11764-s1.doc]

Suppplementary material manuscript: “ADALIMUMAB DELAYS PHOTORECEPTOR CELL DEATH IN A MOUSE MODEL OF RETINAL DEGENERATION”

Cristina Martínez-Fernández de la Cámara, Alberto M Hernández-Pinto, Lorena Olivares-González, Carmen Cuevas-Martí, María Sánchez-Aragó, David Hervás, David Salom, José M. Cuezva, Enrique J de la Rosa, José M Millán, Regina Rodrigo

**a**


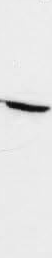


**102**

**76**

**52**

**38**

**31**

**24**

**17**


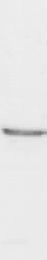


**102**

**76**

**52**

**38**

**31**

**24**

**17**


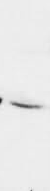


**102**

**76**

**52**

**38**

**31**

**24**

**17**


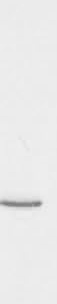


**102**

**76**

**52**

**38**

**31**

**24**

**17**


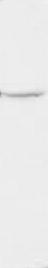


**102**

**76**

**52**

**38**

**31**

**24**

**17**


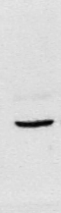


**76**

**52**

**38**

**31**

**24**

**17**

**17**


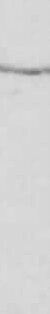


**102**

**76**

**52**

**38**

**31**

**24**

**102**

**76**

**52**

**38**

**31**

**24**


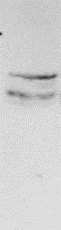


**β-ACTIN**

**GAPDH**

**LDHA**

**SOD1**

**SOD2**

**OPA 1**

**HADHA**

**CATALASE**

**NDUFS3**

**SDHB**

**CORE 2**

**COXII**

**β-F1**

**HSP60**

**G6PDH**


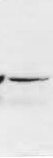


**102**

**76**

**52**

**38**

**31**

**24**

**17**


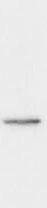


**76**

**52**

**38**

**31**

**24**

**17**


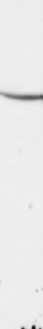


**76**

**52**

**38**

**31**

**24**

**17**


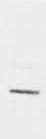


**102**

**76**

**52**

**38**

**31**

**24**

**17**


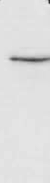


**76**

**52**

**38**

**31**

**24**

**17**

**102**

**76**

**52**

**38**

**31**

**24**

**102**

**76**

**52**

**38**

**31**

**24**

**17**


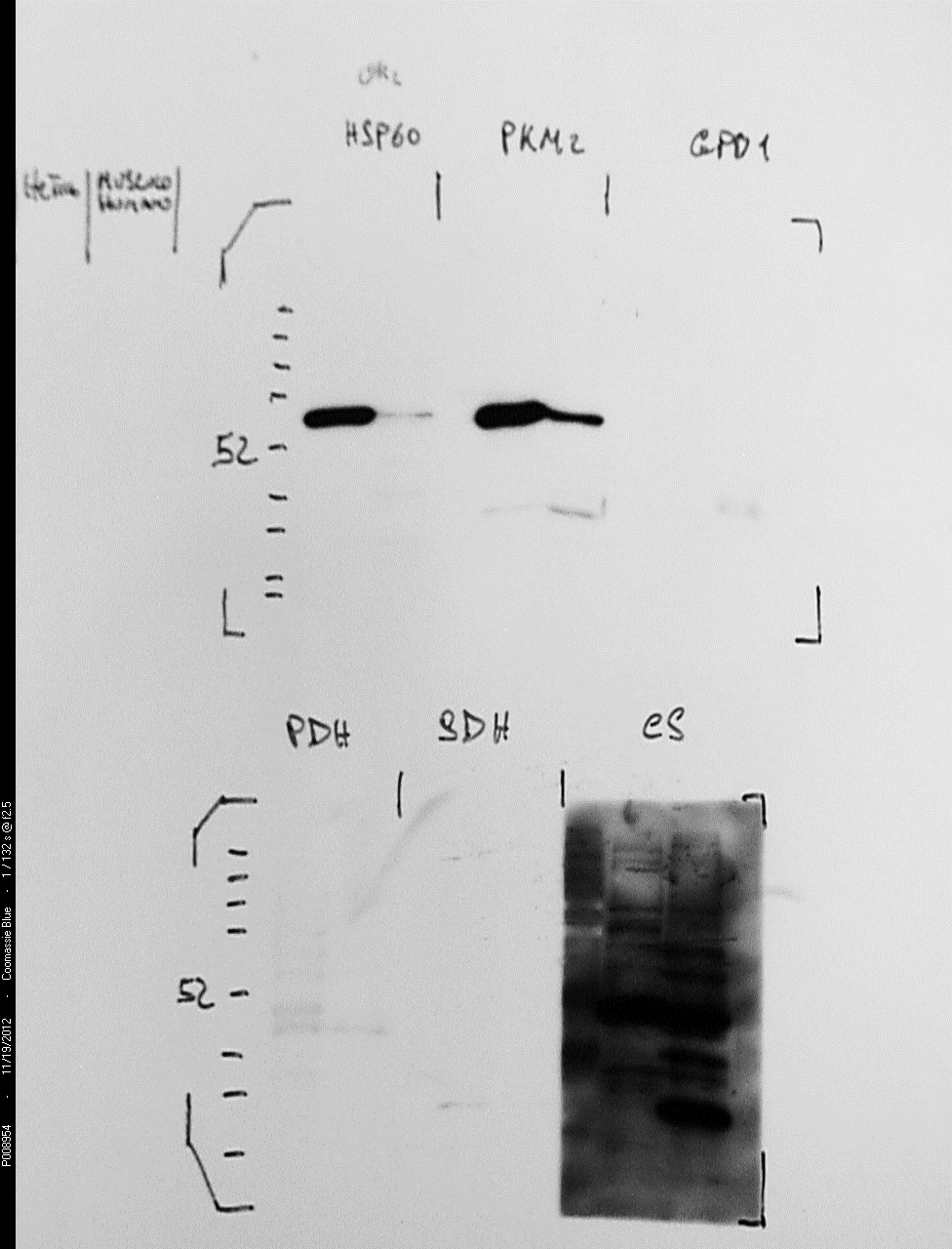

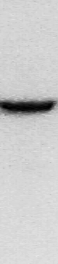


**17**

**b**


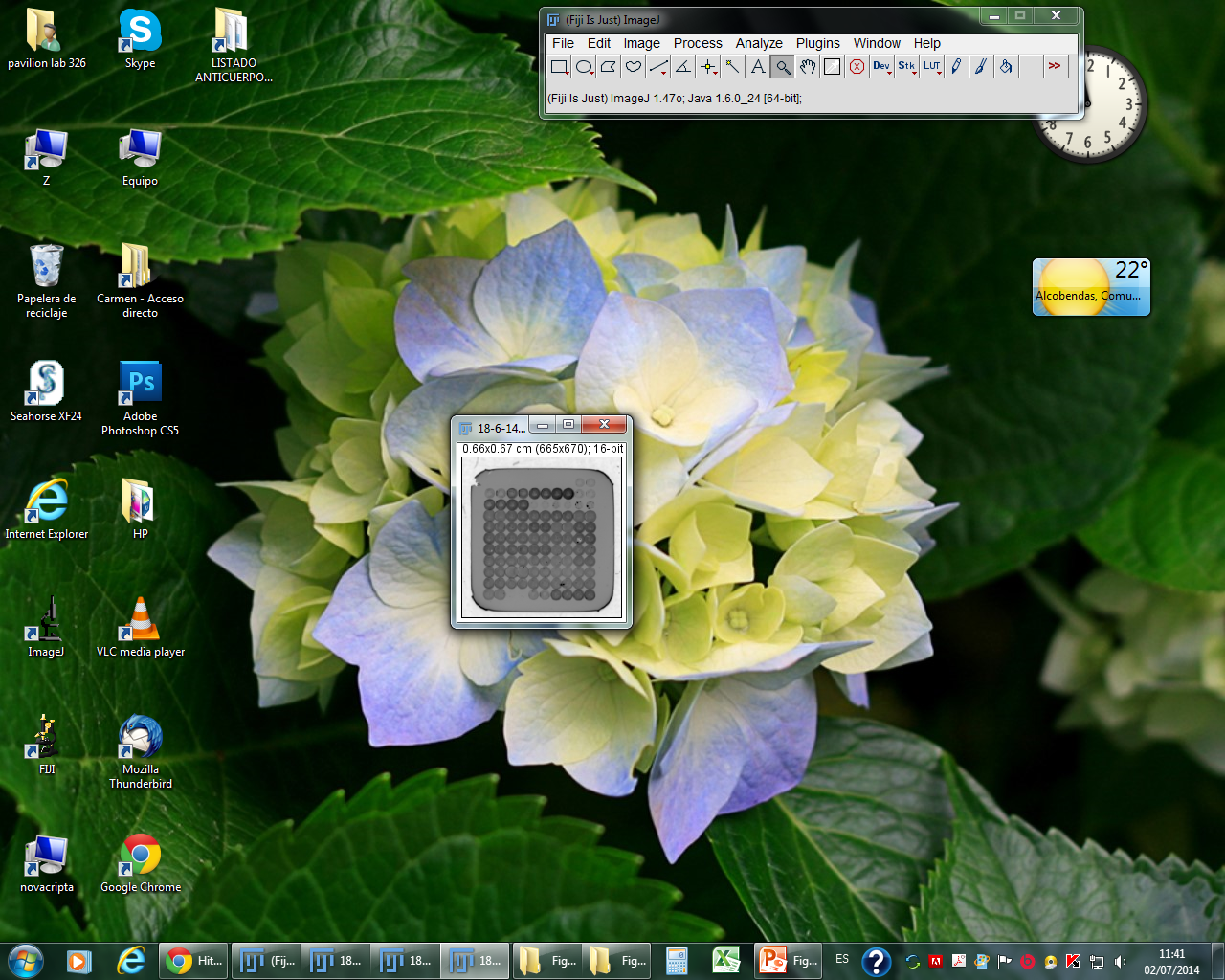

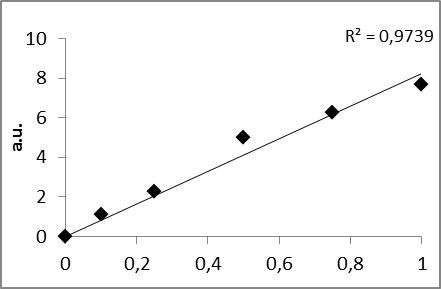


**Figure S1**

Table S1. Mixed linear analysis to evaluate the effect of Adalimumab on retinal degeneration

| **Variable** | **Control estimate** | ***rd10* effect** | **ADA effect** |
| --- | --- | --- | --- |
| **Normalized ONL thickness** | 1.47 [1.27, 1.66] | -0.65 [-0.89, -0.41]  p<0.001 | 0.24 [0.05, 0.44]  p=0.03 |
| **Rows in ONL** | 11.88 [10.02, 13.73] | -6.89 [-9.17, -4.62]  p<0.001 | 2.10 [0.24, 3.96]  p=0.047 |
| **Log(TUNEL positive cells/Normalized ONL thickness)** | -0.11 [-0.91, 0.68] | 2.04 [1.05, 3.04]  p=0.002 | -1.02 [-1.90, -0.13]  p=0.048 |
| **Log(PAR positive cells/Normalized ONL thickness)** | 0.16 [-0.22, 0.5] | 2.9 [2.36, 3.44]  p<0.001 | -2.15 [-2.69, -1.61]  p<0.001 |
| **Migration index of microglia** | 0.15 [0.09, 0.21] | 0.27 [0.19, 0.35]  p<0.001 | -0.15 [-0.23, -0.06]  p=0.006 |
| **Log(GFAP content)** | 14.70 [14.03, 15.37] | 2.09 [1.22, 2.96]  p<0.001 | -0.55 [-1.43, 0.34]  p=0.27 |
| **Log(TNFα expression)** | -0.08 [-0.43, 0.27] | 1.81 [1.30, 2.31]  p<0.001 | -0.99 [-1.66, -0.31]  p=0.006 |
| **TAC** | 131.33 [108.11, 154.55] | -38.61 [-73.05, -4.17]  p=0.031 | 111.63 [73.48, 149.78]  p<0.001 |
| **SOD activity** | 32.92 [27.53, 38.30] | -5.78 [-13.39, 1.83]  p=0.12 | 8.87 [0.36, 17.38]  p=0.042 |
| **CAT activity** | 10.25 [7.43, 13.07] | 5.69 [1.50, 9.88]  p=0.011 | -3.71 [-7.89, 0.48]  p=0.078 |
| **SOD1 content** | 0.73 [0.50, 0.96] | -0.53 [-0.86, -0.20]  p=0.004 | 0.64 [0.30, 0.98]  p=0.001 |
| **SOD2 content** | 0.61 [0.47, 0.75] | 0.10 [-0.12, 0.31]  p=0.35 | 0.19 [-0.03, 0.41]  p=0.087 |
| **CAT content** | 0.39 [0.26, 0.53] | 0.14 [-0.05, 0.83]  p=0.15 | 0.08 [-0.11, 0.27]  p=0.37 |

Note: Mixed linear models were used to assess the association between ADA and the two experimental groups (Control and *rd10*) with the different response variables. Values represent the estimated value for each variable, the confidence interval (between brackets) and the p-value.

**FIGURE LEGEND**

**Figure S1.** **(a)** Validation of the antibodies used for application in RPMM. 40 µg of protein derived from mouse tissues were fractionated on SDS-PAGE gels, blotted against the indicated antibodies and processed for western blotting. Only antibodies that recognize a single protein band of the expected molecular mass (with the exception of OPA1 that recognizes two bands) were used in the study. The migration of molecular mass markers is indicated to the left of the blot. **(b)** Scheme of RPMA printing processed for anti-catalase is shown magnified. One nl samples were spotted in duplicate. Yellow boxed: negative controls of BSA; Blue boxed: standard curve of HCT116 cells; Green boxed: standard curves of OVCAR8 cells (not used in the study); Red boxed: tissue samples from mice retinas. The plot illustrates the linear correlation that exists between the fluorescence intensity (arbitrary units, a.u.) and the amount of catalase in HCT116 cell lysates. Protein concentrations in the samples were calculated according to the fluorescence intensity obtained in the linear plot of HCT116 cells.
